# 256. Characterization of the Placental Proteome during Congenital CMV Infection

**DOI:** 10.1093/ofid/ofad500.328

**Published:** 2023-11-27

**Authors:** Sheila K Farnan, Elise Sintim-Aboagye, Huy Quach, Sohan Punia, Maria Mejia-Plazas, Namisha Verma, Dawn Littlefield, Erica L Johnson, Cosnet Lerato Rametse, Clive Gray, Andrew Norgan, Jerid Robison, Elizabeth Enninga, Rana Chakraborty, Mark R Schleiss

**Affiliations:** UPMC, Pittsburgh, Pennsylvania; Mayo Clinic, Rochester, Minnesota; Mayo Clinic, Rochester, Minnesota; Mayo Clinic, Rochester, Minnesota; Nicklaus Children's Hospital, Miami, Florida; Charles University, Hradec Kralove, Kralovehradecky kraj, Czech Republic; Mayo Clinic, Rochester, Minnesota; Morehouse School of Medicine, Atlanta, Georgia; University of Cape Town, Cape Town, Western Cape, South Africa; Stellenbosch University, Cape Town, Western Cape, South Africa; Mayo Clinic, Rochester, Minnesota; NanoString Technologies, Minneapolis, Minnesota; Mayo Clinic, Rochester, Minnesota; Mayo Clinic, Rochester, Minnesota; University of Minnesota, Minneapolis, MN

## Abstract

**Background:**

The worldwide prevalence of cytomegalovirus (CMV) infection is estimated to be 83-100%.^1,2^ In pregnancy, transmission to the fetus can occur through the placenta with 0.4-2.3% of all newborns affected.^3^ The phenotype of congenital CMV (cCMV) has a spectrum of severity; 10-20% of exposed neonates show severe clinical manifestations.^4^ CMV has a wide range of tropism and can infect epithelial, myeloid, and endothelial cells. However, the cellular mechanisms of cCMV infection, and how they influence disease severity in exposed fetuses during gestation are not fully outlined,^5^ Here, we used the NanoString™ Digital Spatial Profiler (DSP) to characterize the cell-specific proteomic profile in placental tissue from infants with cCMV.

**Methods:**

Placental tissue from 5 CMV-affected and 4 CMV-unaffected pregnancies were retrieved from the Mayo Clinic tissue repository. Immune cells were identified by CD45 positivity and cytotrophoblasts (CTBs) identified by CK7. The cell-specific proteome of these tissues was identified using the NanoString™ immune cell and cell death panels. Data was analyzed by NanoString™ GeoMx technology.

**Results:**

Unsupervised clustering illustrated a distinct proteome in the immune cell compartment of CMV-positive compared to CMV-negative placental tissue (Figure 1). Linear Mixed Model (LMM) analysis revealed that immune cell related proteins were significantly overexpressed in CMV+ tissue as were four proteins associated with cell death: BIM, BCLXL, PARP, neurofibromin (Figure 2). Clustering did not demonstrate a distinct proteome in the CTB compartment (Figure 3), but LMM showed that two immune markers (CD44 and HLA-DR) and a cell death protein (BCL6) were overexpressed by CTBs in CMV+ tissue (Figure 4).

**Figure:**
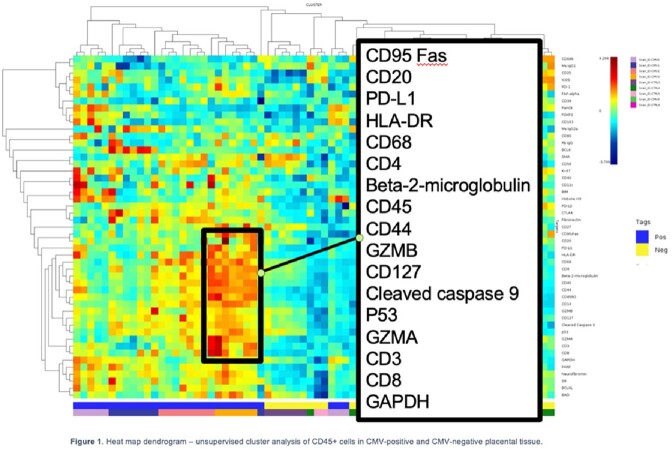

The proteome of immune cells, and to a lesser extent in CTBs in CMV-infected placentae differs from CMV-uninfected placentae with overexpression of proteins associated with immune activation and cell death. With new screening initiatives set to improve identification of cCMV, our results provide a starting point to identify specific biomarkers and immune pathways associated with disease severity. These biomarkers and pathways may serve as novel antiviral targets in the treatment and management of cCMV.

**Figure:**
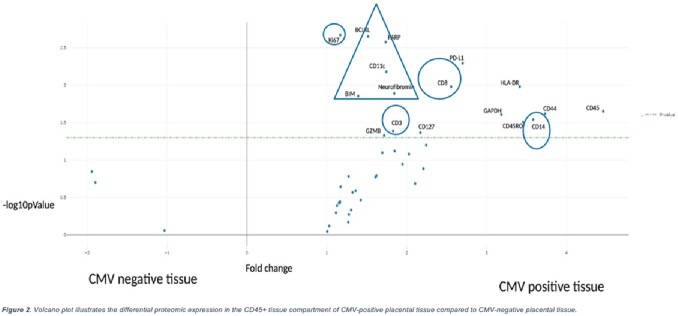

The proteome of immune cells, and to a lesser extent in CTBs in CMV-infected placentae differs from CMV-uninfected placentae with overexpression of proteins associated with immune activation and cell death. With new screening initiatives set to improve identification of cCMV, our results provide a starting point to identify specific biomarkers and immune pathways associated with disease severity. These biomarkers and pathways may serve as novel antiviral targets in the treatment and management of cCMV.

**Figure:**
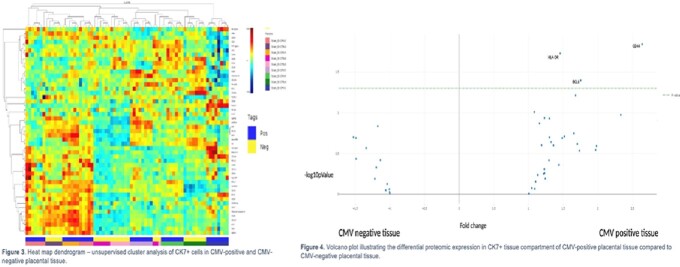

Fig 3 - The proteome of immune cells, and to a lesser extent in CTBs in CMV-infected placentae differs from CMV-uninfected placentae with overexpression of proteins associated with immune activation and cell death. With new screening initiatives set to improve identification of cCMV, our results provide a starting point to identify specific biomarkers and immune pathways associated with disease severity. These biomarkers and pathways may serve as novel antiviral targets in the treatment and management of cCMV. Fig 4 - Volcano plot illustrating the differential proteomic expression in CK7+ tissue compartment of CMV-positive placental tissue compared to CMV-negative placental tissue.

**Conclusion:**

The proteome of immune cells, and to a lesser extent in CTBs in CMV-infected placentae differs from CMV-uninfected placentae with overexpression of proteins associated with immune activation and cell death. With new screening initiatives set to improve identification of cCMV, our results provide a starting point to identify specific biomarkers and immune pathways associated with disease severity. These biomarkers and pathways may serve as novel antiviral targets in the treatment and management of cCMV.

**Disclosures:**

**Mark R. Schleiss, MD**, Moderna Vaccines: Grant Support to my Division but no personal honoraria

